# MicroRNA-200c and microRNA- 141 are regulated by a FOXP3-KAT2B axis and associated with tumor metastasis in breast cancer

**DOI:** 10.1186/s13058-017-0858-x

**Published:** 2017-06-21

**Authors:** Guangxin Zhang, Wei Zhang, Bingjin Li, Erica Stringer-Reasor, Chengjing Chu, Liyan Sun, Sejong Bae, Dongquan Chen, Shi Wei, Kenneth Jiao, Wei-Hsiung Yang, Ranji Cui, Runhua Liu, Lizhong Wang

**Affiliations:** 1 Provincial Key Laboratory on Molecular and Chemical Genetic, The Second Hospital of Jilin University, Changchun, 130041 People’s Republic of China; 2 Department of Genetics, University of Alabama at Birmingham, Birmingham, AL 35294 USA; 3 Chinese Center for Endemic Disease Control, Harbin Medical University, Harbin, 150081 People’s Republic of China; 4 Hematology/Oncology Section, Department of Medicine, University of Alabama at Birmingham, Birmingham, AL 35294 USA; 5 Department of Applied Psychology, Humanities and Management Colleges, Guangdong Medical University, Dongguan, 523808 People’s Republic of China; 6 Division of Preventive Medicine, University of Alabama at Birmingham, Birmingham, AL 35294 USA; 7 Department of Pathology, University of Alabama at Birmingham, Birmingham, AL 35294 USA; 8 Comprehensive Cancer Center, University of Alabama at Birmingham, Birmingham, AL 35294 USA; 9 Department of Biomedical Sciences, Mercer University, Savannah, GA 31404 USA

**Keywords:** microRNA, FOXP3, Breast cancer, Tumor metastasis, Circulation, Exosome

## Abstract

**Background:**

Members of the microRNA (miR)-200 family, which are involved in tumor metastasis, have potential as cancer biomarkers, but their regulatory mechanisms remain elusive.

**Methods:**

We investigated *FOXP3*-inducible breast cancer cells, *Foxp3* heterozygous *Scurfy* mutant (*Foxp3* ^*sf/+*^) female mice, and patients with breast cancer for characterization of the formation and regulation of the miR-200 family in breast cancer cells and circulation. Participants (259), including patients with breast cancer or benign breast tumors, members of breast cancer families, and healthy controls, were assessed for tumor and circulating levels of the miR-200 family.

**Results:**

First, we identified a FOXP3-KAT2B-miR-200c/141 axis in breast cancer cells. Second, aging *Foxp3* ^*sf/+*^ female mice developed spontaneous breast cancers and lung metastases. Levels of miR-200c and miR-141 were lower in *Foxp3* ^*sf/+*^ tumor cells than in normal breast epithelial cells, but plasma levels of miR-200c and miR-141 in the *Foxp3* ^*sf/+*^ mice increased during tumor progression and metastasis. Third, in patients with breast cancer, the levels of miR-200c and 141 were lower in *FOXP3* ^low^ relative to those with *FOXP3* ^high^ breast cancer cells, especially in late-stage and metastatic cancer cells. The levels of miR-200c and miR-141 were higher in plasma from patients with metastatic breast cancer than in plasma from those with localized breast cancer, with benign breast tumors, with a family history of breast cancer, or from healthy controls. Finally, in *Foxp3* ^*sf/+*^ mice, plasma miR-200c and miR-141 appeared to be released from tumor cells.

**Conclusions:**

miR-200c and miR-141 are regulated by a FOXP3-KAT2B axis in breast cancer cells, and circulating levels of miR-200c and miR-141 are potential biomarkers for early detection of breast cancer metastases.

**Electronic supplementary material:**

The online version of this article (doi:10.1186/s13058-017-0858-x) contains supplementary material, which is available to authorized users.

## Background

In the USA, an estimated 231,840 women were diagnosed with breast cancer in 2015, and there were approximately 40,290 deaths [1]. Within 3 years of diagnosis, 10–15% of breast cancers develop distant metastases [2]. Therefore, predictive tests are needed to identify individuals who are at high risk of metastases. To reduce breast cancer metastasis-related morbidity and mortality, the ideal biomarker should be accessible for non-invasive sampling and sensitive enough to detect early onset of tumor metastasis. To date, only a few markers, such as estrogen receptor (ER), progesterone receptor (PR), and human epidermal growth factor receptor 2 (HER2), have been identified as predictors of clinical responses to breast cancer treatments. None of these markers, however, evaluate tumor invasion or provide early detection of tumor metastasis.

MicroRNAs (miRs) are small non-coding RNA molecules that regulate gene expression. In breast cancers, aberrant miR expression influences tumor development and progression [3]. Some miRs are upregulated in breast cancer, whereas others are downregulated, suggesting that miRs may have tumor-specific profiles. MiRs are remarkably stable in the circulation, and in formalin-fixed, paraffin-embedded tissues [4]. Thus, they have potential to serve as breast cancer biomarkers. Circulating miRs, which are present in breast cancer patients, have potential as biomarkers that can be obtained by a minimally invasive procedure [5–10]. Members of the human and mouse miR-200 family (miR-200 s), including two clusters (cluster 1: miR-200b, 200a, and 429, on chromosome 1, and cluster 2: miR-200c and miR-141, on chromosome 12), inhibit the epithelial-mesenchymal transition (EMT) [11, 12] but promote the mesenchymal-epithelial transition (MET) [13, 14], thereby regulating tumor metastasis by a reversible EMT-MET transition. In cancer cells, miR-200 s induce epithelial differentiation by suppressing ZEB1/2 and subsequently increasing E-cadherin expression [11, 12]. Apart from the miR-200-ZEB1/2-E-cadherin axis, miR-200 s inhibit Wnt through *CTNNB1*, NOTCH through *JAG1*, and SNAIL through *SNAI2*, and other direct targets, *FN1*, *MSN*, *NTRK2*, *LEPR,* and *ARHGAP19*, all of which are necessary for tumor metastasis [15–17]. Decreased levels of miR-200 s in tumor cells have been implicated in the invasion and metastasis of breast cancer [11, 12, 18], but, in preclinical models, restoration of miR-200c reduced metastases [19], suggesting that the miR-200 s function as tumor suppressors. Conversely, some studies suggest that, in cancer cells, miR-200 s promote tumor metastasis through promotion of tumor colonization at metastatic sites [13, 14]. In murine cancers and human xenograft models, miR-200-expressing tumor cells and extracellular vesicles from these tumor cells promote breast cancer metastasis and confer the capacity for these cells to colonize distant tissues in an miR-200-dependent manner [20]. Further, high levels of circulating miR-200 s in breast cancer patients are associated with increased numbers of circulating tumor cells (CTCs) [21], which are a predictor of metastasis up to 2 years prior to clinical diagnosis [22] and of shorter brain-metastasis-free survival [23]. Thus, circulating miR-200 s are promising biomarkers for breast cancer metastasis. However, the cellular origin, mechanism of release, and function of circulating miR-200 s during tumor progression and metastasis remain elusive.

FOXP3 functions as the master regulator in the development and function of regulatory T cells [24]. Our group found that FOXP3 is also a breast epithelial cell-intrinsic tumor suppressor [25–27]. Unlike normal breast epithelial cells, 60–80% of human breast cancer cells lack nuclear expression of FOXP3 [25, 26, 28, 29]. Aging female mice with a heterozygous *Scurfy* (*sf*) mutation of *Foxp3* (*Foxp3* ^sf/+^), which causes a loss of *Foxp3* expression, frequently develop spontaneous breast cancers (ER^+^, 14/18; PR^+^, 12/18; ErbB2^+^, 18/18) after 1 year of age, and 40% of these mice with primary breast tumors also develop lung metastases [26]. Thus, this mouse model is appropriate for finding and validating breast cancer biomarkers and for evaluating their cellular origin, identifying their mechanism of release, and assessing the function of circulating miRs during tumor progression and metastasis. In the present work, we explored the relevance of FOXP3-mediated transcriptional regulation of miR-200 s in breast cancer cells in mice and humans. We also investigated the cellular origin, mechanism of release, and function of plasma miR-200 s in breast cancer cells and in mouse models.

## Methods

### Cell lines, antibodies, and reagents

Breast cancer cell lines MCF7, T47D, BT474, and MDA-MB-468 were obtained from the American Type Culture Collection (Manassas, VA, USA). Cell lines were authenticated by examination of morphology and growth characteristics and were confirmed to be mycoplasma-free. A short tandem-repeat analysis for DNA fingerprinting was also used to verify the human cell lines. Green fluorescent protein (GFP) and FOXP3/GFP-Tet-off MCF7 cells were established and maintained in doxycycline (Dox, 10 μg/ml) as described previously [25, 30]. Specific primary antibodies were used to detect the following proteins: FOXP3 (ab450, 1:2,000, ABCAM, Cambridge, MA, USA), PCAF (KAT2B) (C14G9, 1:1,000, Cell Signaling Technology, Danvers, MA, USA), and PITX2 (ab98297, 1:2,000, ABCAM). The pEF1-FOXP3-V5 vector [31] or empty pEF1 vector was transfected into cells using FuGENE6 (Promega, Madison, WI, USA). *KAT2B* and *PITX2* small interfering RNAs (siRNAs) are listed in Additional file 1: Table S1.

### Experimental animals

Mice with an *sf* mutation in the *Foxp3* locus were purchased from the Jackson Laboratory (Bar Harbor, ME, USA). The *sf* mouse has a spontaneous *Foxp3* frameshift mutation, resulting in loss of FOXP3 function. Homozygous or hemizygous *sf* mice generally die within 2 weeks after birth, but heterozygous scurfy female mice have a normal life span. In our previous studies [26], we established the *Foxp3* ^*sf/+*^ mouse model, which was used here for monitoring the mechanisms involved in the progression and metastasis of breast cancers. These mice have a BALB/c background. F1 *Foxp3* ^*sf/+*^ female mice of appropriate genotypes were used. All animal experiments were conducted in accordance with accepted standards of animal care and approved by the Institutional Animal Care and Use Committee of University of Alabama at Birmingham (UAB).

### Human subjects

The plasma and blood samples from 259 human subjects, including 114 patients with breast cancer, 30 patients with benign breast tumors, 21 women with a family history of breast cancer, and 94 healthy women were obtained from the UAB Tissue Collection and Banking Facility (Table 1). Patient demographics and tumor clinicopathologic details are shown in Table 1. The pathologic stage of breast cancer at the time of diagnosis was determined by use of the tumor-node-metastasis (TNM) system. Tumor grading was that corresponding to grade 1 (well-differentiated), grade 2 (moderately differentiated), or grade 3 (poorly differentiated) tumors. All patients with distant metastasis were diagnosed by follow up after surgery. Normal female controls (identified by a routine health visit) were matched with patients for age, race, reproductive status, region of residence, and duration of plasma storage. Only plasma obtained prior to any clinical treatment was used.

**Table 1:** Characteristics of human subjects

	First cohort				Second cohort	
Categories	BC^a^	Benign^b^	Family^c^	Control^d^	BC^a^	Control^d^
Total number	39	30	21	44	75	50
Recruiting time, years	2004–2014	2004–2014	2004–2014	2004–2014	2004–2014	2004–2014
Median age (range), years	51 (30–73)	51 (40–63)	47 (33–71)	50 (36–70)	52 (32–75)	52 (37–71)
Race						
Caucasian	26	28	20	36	75	50
African-American	11	1	1	7	0	0
Other	2	1	0	1	0	0
ER status						
Positive	26				46	
Negative	4				29	
Unknown	9				0	
PR status						
Positive	24				41	
Negative	5				34	
Unknown	10				0	
HER2 status						
Positive	6				28	
Negative	25				47	
Unknown	8				0	
Histological subtype						
Ductal	30				58	
Lobular	2				9	
Both	2				0	
Unclassified	5				8	
Tumor grade						
Well	2				4	
Moderate	13				38	
Poor	17				33	
Unknown	7				0	
Tumor stage						
Localized						
DCIS (pTis)	6				0	
Early (pT1-2N0M0)	15				42	
Advanced (pT3-4N0M0)	4				8	
Metastatic						
Regional (pT1-4 N1-3 M0)	11				15	
Distant (pT1-4 M1)	3				10	

*ER* estrogen receptor, *PR* progesterone receptor, *HER2* human epidermal growth factor receptor, *DCIS* ductal carcinoma in situ, *T* tumor, *N* node, *M* metastasis. ^a^Patients with breast cancer. ^b^Patients with benign breast tumor. ^c^Normal healthy women with family history of breast cancer. ^d^Normal healthy women without family history of breast cancer

Two independent cohorts of human subjects were divided for assessment and validation of the miR-200 family as potential biomarkers. For the first cohort, we obtained plasma from 134 human subjects, including 33 patients with invasive breast cancer (19 with pT1-4, 11 with N1-3, and 3 with M1), 6 patients with non-invasive breast cancer (ductal carcinoma in situ (DCIS), pTis), 30 patients with benign breast tumors, 21 women with a family history of breast cancer, and 44 healthy female controls. In the second cohort, we obtained the plasma from 125 human subjects in a separate Caucasian population, including 50 patients with local breast cancer (42 with pT1-2N0M0 and 8 with pT3-4N0M0) and 25 patients with metastatic breast cancer (15 with only lymph node involvement (N1-3) after surgery, and 10 with distant metastatic disease (i.e., lungs, liver, bones) (M1) diagnosed after surgery), and 50 normal healthy women. In addition, we obtained blood cells from 30 of 125 human subjects, including 10 patients with local breast cancer (pT1-4N0M0) and 10 patients with metastatic breast cancer (distant metastasis after surgery), and 50 healthy female controls. The ER/PR/HER2 status in patients with breast cancer was: ER, 46 positive and 29 negative patients; PR, 41 positive and 34 negative patients; HER2, 28 positive and 47 negative patients. Tumor grades for patients in breast cancer were well-differentiated (4 patients), moderately differentiated (38 patients), and poorly differentiated (33 patients). Tumor types in patients with breast cancer were invasive ductal carcinoma (58 patients), invasive lobular carcinoma (9 patients), and unclassified type (8 patients).

### Sample collection

Blood from mice and humans was collected in EDTA tubes (BD Biosciences). All samples for isolation of plasma and blood cells were centrifugally processed within 4 hours of collection. To avoid the release of miRs from blood cells during the coagulation process [32], miRs in plasma were assessed. Cell-free plasma and blood cells were stored at −80 °C until analysis.

### Laser capture microdissection

Laser capture microdissection was performed as described previously [33]. For analysis of gene expression, cells (5 × 10^3^) were microdissected from target tissues.

### Exosome isolation

Cell culture medium and plasma was centrifuged at 300 × g for 10 minutes to clear cells and large debris. The supernatant was centrifuged at 2000 × g for 20 minutes and then at 10,000 × g for 30 minutes to remove residual membranous debris. The remaining supernatant was subjected to ultracentrifugation at 100,000 × g for 70–120 minutes to pellet the exosomes [34, 35].

### RNA isolation

For isolation of RNA, 200 μl of plasma or 2 μg of purified exosomes in 200 μl PBS were thawed on ice and lysed with an equal volume of 2x Denaturing Solution (Life Technologies). To normalize sample-to-sample variation in plasma or exosomal RNA isolation, 25 fmol of synthetic *Caenorhabditis elegans* miR cel-miR-39 (QIAGEN, Valencia, CA, USA) was added to each denatured sample [4]. Total RNA was extracted using miRNeasy Serum/Plasma Kits (QIAGEN). RNA was isolated from blood cells and cultured cells by the Trizol (Life Technologies) method.

### TaqMan miR assay

Levels of mature miR-200 s in tumor cells, cultured cells, and blood cells were assessed by use of TaqMan MicroRNA Assays (Life Technologies) as described previously [31, 33]. The average relative amounts were determined using the comparative method (2^-ΔCt^) against endogenous *RNU6B* (for humans) or *snoRNA202* (for mice) controls.

### Nest-quantitative PCR (qPCR) analysis

Due to the low amounts of mature miR-200 s in plasma and exosomes, nest-qPCR analyses were performed to measure mature miR-200 s. Briefly, 5 μl of RNA in 20 μl reactions was reverse-transcribed using the miScript II RT Kit (QIAGEN). cDNA (2 μl) was added to 20-μl reactions for pre-amplification PCR as described previously [4]. Then, 2-μl portions of the PCR products were used as templates for real-time PCR with a LightCycler 480 Real Time PCR System (Roche Applied Sciences, Indianapolis, IN, USA) with miScript SYBR Green PCR kits (QIAGEN) at 95 °C for 2 minutes, followed by 40 cycles of 95 °C for 15 sec and 60 °C for 1 minute. The relative quantities of miRs in plasma were determined with an adjusted cycle threshold (aCt) value against the spiked-in control cel-miR-39 (QIAGEN) as described previously [21]. The relative quantities in exosomes were determined by the comparative method (2^-ΔCt^) against the spiked-in control cel-miR-39 (QIAGEN). The nest-qPCR primers are listed in Additional file 1: Table S1.

### Western blots and quantitative chromatin immunoprecipitation (ChIP) assays

Western blotting was performed as previously described [25, 26, 31]. For nuclear proteins, the cells were first incubated in buffer A (10 mmol/L HEPES (pH 7.8), 10 mmol/L KCl, 2 mmol/L MgCl_2_, 0.1 mmol/L EDTA, 1% NP40, and protease inhibitors), and the pellet was suspended in buffer B (50 mmol/L HEPES (pH 7.8), 300 mmol/L NaCl, 50 mmol/L KCl, 0.1 mmol/L EDTA, 10% (v/v) glycerol, and protease inhibitors). ChIP assays were accomplished according to our previous reports [25, 26, 31]. The FOXP3-expressing tet-off cells were sonicated and fixed with 1% paraformaldehyde. Anti-FOXP3 and anti-IgG (Santa Cruz Biotechnology) antibodies were used to pull down chromatin associated with FOXP3. The amounts of specific DNA fragments were quantitated by real-time PCR and normalized against the genomic DNA preparations from the same cells. The ChIP qPCR primers are listed in Additional file 1: Table S1.

### Statistical analyses

Continuous variables were summarized using sample size, mean, standard deviation (SD), median, and minimum and maximum values. Categorical data were summarized using frequency and proportion. The distribution of data for each group was evaluated using the one-sample Kolmogorov-Smirnov test. In samples with normal distributions, the means of the variables were compared between two groups using the two-tailed *t* test. In samples with non-normal distributions, the medians of the variable were compared between two groups using the Mann-Whitney test. One-way and two-way analysis of variance (ANOVA) was used to test for overall differences, followed by the protected least significant difference test for differences between groups. The receiver operating characteristic (ROC) curve was used to assess the diagnostic accuracy of each miR, and the sensitivity and specificity of the optimum cutoff point was defined as those values that maximized the area under the curve (AUC). All data were entered into an Access database using Excel 2013 and analyzed using SPSS (version 20; IBM, Armonk, NY, USA), StatView (version 5.0.1), and SAS (SAS Institute Inc., Cary, NC, USA).

## Results

### FOXP3 induces miR-200c and miR-141 in human breast cancer cells

**Fig. 1:**
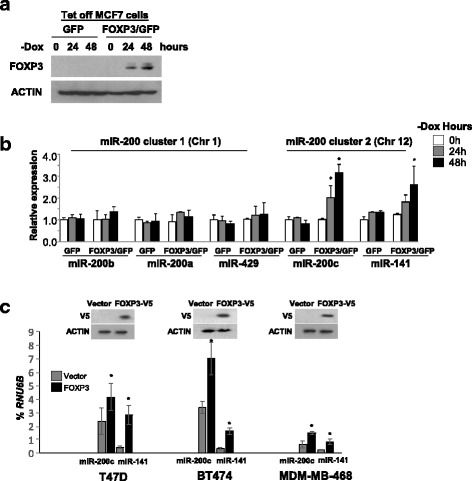
FOXP3 induces miR-200c and miR-141 in breast cancer cells. **a** FOXP3 protein expression in green fluorescent protein (*GFP*) and FOXP3/GFP-Tet-off MCF7 cells was measured by western blotting. *-Dox* doxycycline removed from the culture medium. **b** Relative quantification (by quantitative (q)PCR) of miR-200 clusters 1 and 2 in FOXP3/GFP-Tet-off MCF7 cells without Dox at 0, 24, and 48 h. The concentrations of miR-200 s at 0 h were used as a reference. *Chr* chromosome. Data are presented as means ± SD. *p < 0.05 vs. 0-h group, analysis of variance followed by the protected least significant difference test for differences between groups. **c** Quantification of miR-200 s (by qPCR) as a percentage of *RUN6B* expression in T47D (*left*), BT474 (*middle*), and MDA-MB-468 (*right*) cells at 48 h after transfection. Representative western blot analyses for FOXP3 expression in FOXP3-V5-transfected cells (*inset* in each panel). Data are presented as the means ± SD. *p < 0.05 vs. vector group, two-tailed *t* test. All experiments were repeated three times

The FOXP3/GFP-Tet-off MCF-7 cell lines (ER^+^, PR^+^, HER2^-^) were used to express FOXP3 in the absence of Dox (Fig. 1a). Levels of miR-200 s in the FOXP3/GFP-Tet-off MCF-7 cell lines were assessed by use of qPCR at 24 and 48 hours after FOXP3 induction. As shown, miRs-200c and miR-141 at miR-200 cluster 2 (2.0-fold to 3.2-fold miR-200c induction; 1.8-fold to 2.6-fold miR-141 induction), but not miRs-200b, 200a, and 429 at miR-200 cluster 1, were induced after FOXP3 induction (Fig. 1b). These observations were confirmed with FOXP3-transfected T47D (ER^+^, PR^+^, HER2^-^), BT474 (ER^+^, PR^+^, HER2^+^), and MDA-MB-468 (ER^-^, PR^-^, HER2^-^) cells at 48 hours after transfection (1.8-fold to 2.4-fold miR-200c induction, 3.6-fold to 6.7-fold miR-141 induction) (Fig. 1c). Since endogenous *FOXP3* is expressed in the normal immortalized human epithelial cell line, MCF10A [31], we knocked down *FOXP3* in MCF10A cells by short hairpin RNAs [31] and found that expressions of miR-200c and miR-141 in the cells were decreased after *FOXP3* silencing (Additional file 1: Figure S1A-B).

To determine if miR-200 is expressed in human breast cancers, the breast invasive carcinoma (*n* = 1100) expression dataset (RNAseqV2, UNC) from NCI The Cancer Genome Atlas (TCGA) was examined. In total, 542 samples were available for analyses of expression of both miR-200 s and *FOXP3*. These were divided into two subgroups (*FOXP3* ^*l*ow^ and *FOXP3* ^high^) by a medium value of *FOXP3* expression in tumor samples. High levels of all miR-200 s were present in FOXP3^high^ tumors relative to those in FOXP3^low^ tumors (Additional file 1: Figure S2A). Since endogenous expression of *FOXP3* in FOXP3^+^ tumor-infiltrating lymphocytes (TILs) is more than 100-fold higher than in breast epithelial cells [36, 37], FOXP3^+^ TILs may contribute to the expression of *FOXP3* in tumors. Most FOXP3^+^ TILs, but not epithelial cells, express *CD25* (*IL2RA*), allowing further stratification of patients by the extent of *CD25* expression in tumors (Additional file 1: Figure S2B). To avoid the potential effect of FOXP3^+^ TILs, we selected the *CD25* ^low^ tumors, indicative of few FOXP3^+^ TILs, to assess the association between expression of *FOXP3* and miR-200 s in the TCGA breast cancers (Additional file 1: Figure S2C). Likewise, high levels of all miR-200 s were validated in *FOXP3* ^high^ tumors relative to those in *FOXP3* ^low^ tumors (Additional file 1: Figure S2C).

### Molecular mechanism for transcriptional regulation of miR-200c and miR-141 by FOXP3 in breast cancer cells

**Fig. 2:**
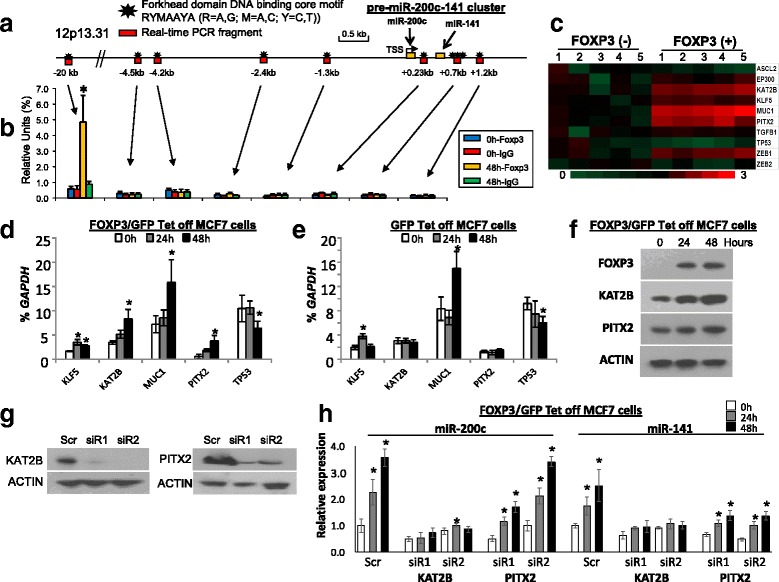
FOXP3 induces the transcriptional activity of miR-200c and miR-141 in breast cancer cells through KAT2B. **a** Location of human miR-200c and miR-141 on chromosome 12. Forkhead consensus motifs are indicated as *black stars*, and PCR-amplified DNA fragments are depicted as *red boxes* below *black stars*. **b** Amounts of DNA precipitated, expressed as a percentage of the total input DNA, as determined by chromatin immunoprecipitation (ChIP) analyses of FOXP3-binding sites in the promoter region of miR-200c and miR-141 in FOXP3/GFP-Tet-off MCF7 cells without doxycycline (Dox) at 0 and 48 h. A FOXP3-binding locus up to 20 kb upstream of miR-200c/141, as shown in our ChIP-seq data (Additional file 1: Figure S3B), was used as a positive control for the FOXP3 ChIP assay. **c** Heat map depiction of alterations in FOXP3 target gene expression in FOXP3/GFP-Tet-off MCF7 cells without Dox at 0 and 48 h generated from Affymetrix Human U133 Plus 2.0 microarrays. The microarray data were submitted to EMBL-EBI (accession number E-MTAB-73). **d**, **e** mRNA expression of FOXP3 target genes (measured by quantitative (q)PCR) as a percentage of *GAPDH* expression in FOXP3/GFP-Tet-off MCF7 cells without Dox at 0, 24, and 48 h. Data are presented as means ± SD. *p < 0.05 vs. 0-h group (one-way analysis of variance (ANOVA) followed by the protected least significant difference test). **f** Western blot analyses showing protein expression of *FOXP3* and its target genes before and after FOXP3 induction in FOXP3/GFP-Tet-off MCF7 cells. **g** Western blot analyses showing protein expressions of KAT2B and PITX2 in scramble (*Scr*) or siRNA-transfected cells. **h** Relative quantification of miR-200c and miR-141 (by qPCR) as percentages of *RNU6B* in FOXP3/GFP-Tet-off MCF7 cells with scramble or siRNA of FOXP3 target genes at 0, 24, and 48 h. All data in each group were normalized to the scramble control at 0 h. Data are presented as the means ± SD of triplicates. *p < 0.05 vs. 0-h group (one-way ANOVA followed by the protected least-significant difference test). All experiments were repeated three times

According to our previous ChIP-sequencing data [30], the binding signals of FOXP3 are close (-3.4 kb) to the locus at non-regulated miR-200 cluster 1 (miR-200a/b/429) but distal (-20 kb) to the locus at FOXP3-regulated miR-200 cluster 2 (miR-200c/141) in FOXP3/GFP-Tet-off MCF-7 cells (Additional file 1: Figure S3A-B). In the present study, no FOXP3-binding signal was evident at the miR-200 cluster 2 locus (-5 kb/+2 kb) as determined by ChIP assays with qPCR analyses (Fig. 2a, b). These data indicate that in MCF-7 cells, miR-200c and miR-141 are regulated indirectly by FOXP3. Furthermore, the literature was reviewed to identify 10 candidates of transcriptional regulators of miR-200c and miR-141: ASCL2 [38], EP300 [39], KAT2B [39], KLF5 [40], MUC1 [41], PITX2 [42], TGFβ1 [43], TP53 [44], and ZEB1/2 [45]. Expressions of these genes in FOXP3/GFP-Tet-off MCF7 cells were analyzed before and after FOXP3 induction (Fig. 2c) using our previous microarray data with ChIP-sequencing data [30, 31]. To confirm promising candidates, qPCR analyses were performed for mRNA expression of all candidate genes. After FOXP3 induction in FOXP3/GFP-Tet-off MCF7 cells, five candidate genes (*KAT2B*, *KLF5*, *MUC1*, *PITX2*, and *TP53*) had a >1.5-fold change in gene expression (Fig. 2d), but, in the GFP-Tet-off MCF7 control cells, two of these five candidate genes (*KAT2B* and *PITX2*) had no significant change (<1.5-fold, Fig. 2e). The downregulation of KAT2B and PITX2 proteins in response to FOXP3 induction were validated by western blots (Fig. 2f). Thus, in FOXP3-mediated transcriptional regulation of miR-200c and miR-141, *KAT2B* and *PITX2* appeared to be coordinators between FOXP3 and miR-200c and miR-141. To evaluate this possibility, siRNAs were used to knock down *KAT2B* and *PITX2* (Fig. 2g). Of note, miR-200c/141 were reduced after KAT2B or PITX2 knockdown and were then induced by FOXP3 in the PITX2-silenced cells, but no significant changes were evident after FOXP3 induction in the KAT2B-silenced cells (Fig. 2h). The results established that *KAT2B*, but not *PITX2*, is required for FOXP3-mediated transcriptional induction of miR-200c and miR-141. However, the effect of KAT2B knockdown on the miR-200 cluster 1 miRNAs was not observed in FOXP3/GFP-Tet-off MCF7 cells (Additional file 1: Figure S4). In addition, we analyzed the FOXP3 ChiP-seq data and identified direct binding signals of FOXP3 in the promoter region of *KAT2B* in FOXP3/GFP tet-off cells (Additional file 1: Figure S3C).

### During tumor progression, the amounts of miR-200c and miR-141 are low in primary breast cancer cells but high in circulating plasma

**Fig. 3:**
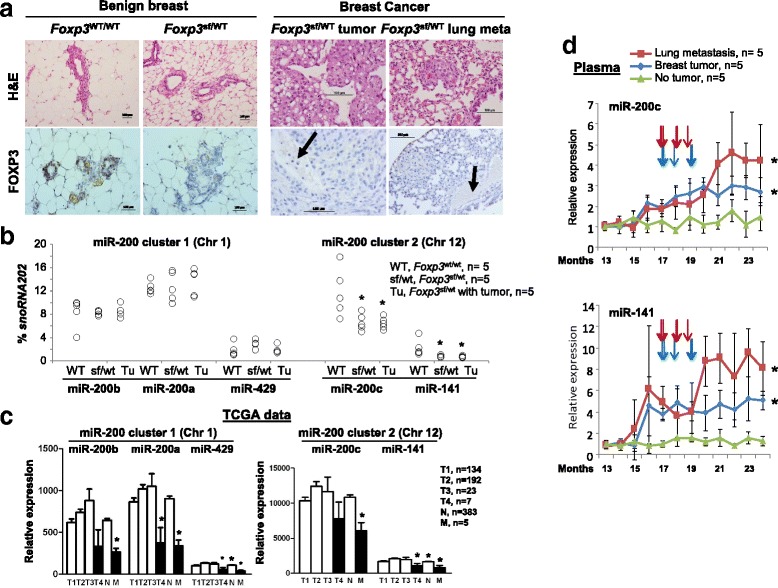
Changes in miR-200 s in tumor cells and plasma during breast cancer progression. **a** Representative hematoxylin and eosin (*H&E*) and FOXP3 immunohistochemical staining of breast tissues, breast tumors, and lung metastases in mice. *Meta* metastasis, *WT* wild-type, *Sf* scurfy, *Tu* breast tumor. *Black arrows* indicate the vasculature. **b** Levels of cluster 1 and cluster 2 miR-200 s (measured by quantitative (q)PCR) as percentages of *snoRNA202* expression in microdissected breast epithelial cells and tumor cells. *p < 0.05 vs. WT group, two-tailed *t* test. *Chr* chromosome. **c** Concentrations of cluster 1 and cluster 2 miR-200 s in human breast cancer samples at various tumor stages (tumor-node-metastasis (*TNM*) staging classifies cancers based on T1-T4, N, and M), as determined from data for 271 patients listed in the NCI The Cancer Genome Atlas (TCGA). Data are presented as the means ± SD. *p < 0.05 vs. T1-3 group, two-tailed *t* test. **d** Plasma levels of miR-200c and miR-141 (determined by nest-qPCR) during tumor progression in *Foxp3* ^sf/+^ female mice. The time points of breast tumor development are indicated by *vertical arrows* (*red arrows* and *blue arrows* for metastatic and non-metastatic mice, respectively). Tumor progression was determined by the size and distant metastasis of tumors. The relative amounts were calculated using the 2^-∆Ct^ against the cycle threshold (Ct) value at 1 year of age (*p < 0.05 vs. no tumor group; two-way analysis of variance). The time points of tumor development, assessed by visual observation, are indicated by *vertical arrows*. All experiments were repeated three times

MiR-200c is 100% conserved between mice and humans, and miR-141 differs by one nucleotide in *5p*, demonstrating that miRs-200c and miR-141 are highly conserved between mice and humans. To validate our observation in breast cancer cells, we used heterozygous *Foxp3* ^sf/+^ breast cancer mice to analyze the regulation of mouse miR-200 s during tumor progression. By 1.5 years of age, approximately 40% of untreated *Foxp3* ^sf/+^ female mice develop tumors, and 40% of those with primary breast tumors experience lung metastases [26]. For each group, 20 mice at 2 years of age were killed, except for those that died or were sacrificed early due to tumor size (>1.0 cm^3^) or extensive tumor metastasis. Histologic examinations and expression analyses were made for five *Foxp3* ^+/+^ wild-type mice, five *Foxp3* ^sf/+^ mice without tumors, five mice with breast tumors only, and five mice with both breast tumors and lung metastases (Fig. 3a). Our previous study demonstrated that FOXP3 protein is reduced in *Foxp3* ^sf/+^ breast epithelial cells [26]. By use of laser-capture microdissection, *Foxp3* ^+/+^ or *Foxp3* ^sf/+^ breast epithelial cells and *Foxp3* ^sf/+^ breast cancer cells were obtained from these mice, and the levels of miR-200 s in the microdissected cells were measured. Levels of miR-200c and miR-141, but not those of miR-200b, miR-200a, and miR-429, were reduced in the *Foxp3* ^sf/+^ breast epithelial cells and tumor cells compared with wild-type breast epithelial cells (Fig. 3b). These data indicate that miR-200c and miR-141 at miR-200 cluster 2 are downstream targets of FOXP3. Furthermore, the miR expression dataset for invasive breast carcinomas, downloaded from the NCI TCGA, was analyzed with tumor stages. In total, 744 patients were available for analyses of expression of miR-200 s. Most miR-200 s were downregulated in late-stage primary tumors, but all miR-200 s were downregulated in the primary tumors with metastasis (Fig. 3c). Results of studies of human breast cancers support this observation [5, 21, 46]. In breast cancer cells, however, expression of miR-200 s was not related to ER/PR/HER2 status (Additional file 1: Figure S5A-C).

To determine the levels of miR-200 s in circulation during tumor progression, plasma was collected monthly from *Foxp3* ^sf/+^ female mice after 1 year of age. Plasma miR-200 s levels were measured at 12 time points by a nest-qPCR analysis. During tumor progression in the *Foxp3* ^sf/+^ female mice, there were increased levels of plasma miR-200c and miR-141 at miR-200 cluster 2 (Fig. 3d, the time points of breast tumor development are indicated by vertical arrows) but not at miR-200 cluster 1 (Additional file 1: Figure S6). Furthermore, plasma miR-200c and miR-141 levels were elevated during tumor metastasis (Fig. 3d).

### In patients with breast cancer, high levels of plasma miR-200c and miR-141 are associated with tumor metastasis

**Fig. 4:**
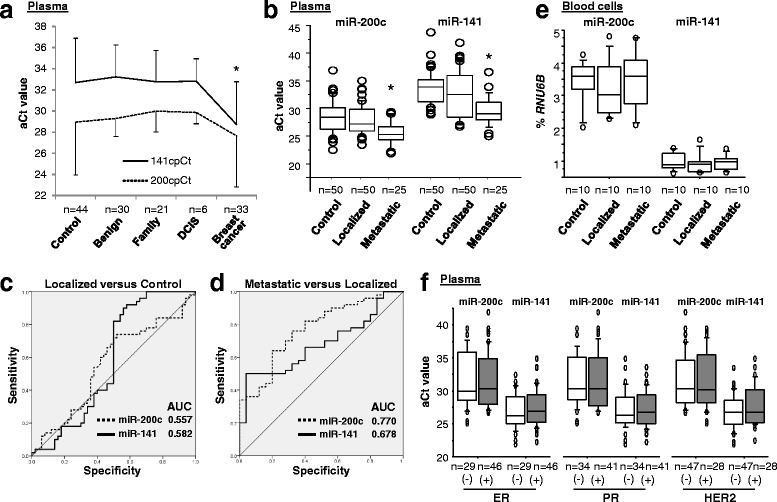
Plasma levels of miR-200c and miR-141 are elevated in patients with metastatic breast cancer. **a** Plasma levels of miR-200c and miR-141 presented as adjusted PCR cycle threshold (*aCt*) values for normal female controls, patients with benign breast tumors, those with a family history of breast cancer, patients with breast ductal carcinoma in situ (*DCIS*), and patients with breast cancer. The aCt value of each miR was normalized to cel-miR-39 as described previously [4, 21]. In plasma levels of miR-141, *p < 0.05 breast cancer vs. other groups (one-way analysis of variance (ANOVA) followed by the protected least significant difference test). **b** Plasma miR-200c and miR-141 levels represented as aCt values for patients with localized breast cancer, patients with metastatic breast cancer, and normal female controls. *p < 0.05 disease vs. control group (one-way ANOVA followed by the protected least significant difference test). **c**, **d** Sensitivity and specificity of plasma miR-200c and miR-141 for patients with localized breast cancer relative to normal female controls and patients with metastatic breast cancer relative to patients with localized breast cancer. Receiver operating characteristic curves are shown for miR-200c (*dotted line*) and miR-141 (*solid line*). *AUC* area under the curve. **e** Llevels of miR-200c and miR-141 in blood cells presented as percentages of *RUN6B* expression for patients with localized breast cancer, patients with metastatic breast cancer, and normal female controls. **f** Plasma miR-200c and miR-141 levels presented as aCt values for breast cancer patients with estrogen receptor (*ER*), progesterone receptor (*PR*), and human epidermal growth factor receptor 2 (*HER2*) status. Data for all patients in this figure are not included in the The Cancer Genome Atlas datasets. All experiments were repeated three times

To determine if expression patterns in human breast cancers reflect the data from mice, the levels of plasma miR-200c and miR-141 were examined by nest-qPCR analysis of a breast cancer population. Demographic and other variables, including age, ethnicity, ER/PR/HER2 status, and clinical factors are summarized in Table 1. The plasma levels of miR-200c and miR-141 were determined for 33 patients diagnosed with breast cancer (19 with pT1-4, 11 with N1-3, and 3 with M1), 6 patients with ductal carcinoma in situ (DCIS), 30 patients with benign breast tumors, 21 women with a family history of breast cancer, and 44 healthy female controls. For patients with invasive breast cancer, plasma levels of miR-141, but not miR-200c, were higher (a lower adjusted Ct value correlated with a higher level) than levels for the other groups examined (Fig. 4a). There was no significant difference in plasma levels of miR-200c and miR-141 between patients with DCIS and healthy controls. There was also no significant difference in plasma miR-200c and miR-141 levels between healthy women with and without a family history of breast cancer, suggesting that plasma levels of miR-200c and miR-141 are not hereditary.

For an independent Caucasian cohort, the plasma levels of miR-200c and miR-141 were examined for 50 patients with local breast cancer (42 with pT1-2N0M0 and 8 with pT3-4N0M0) and 25 patients with metastatic breast cancer (15 with only lymph node involvement (N1-3) after surgery, and 10 with distant metastatic disease (i.e., lungs, liver, bones) (M1) diagnosed after surgery). Also analyzed were 50 age-matched and ethnicity-matched healthy female controls. The *t* tests confirmed significantly higher levels (lower adjusted Ct values) of plasma miR-200c and miR-141 in metastatic breast cancers compared to breast cancers localized to breast tissue only (miR-200c: 5.5-fold, *p* < 0.001; miR-141: 7.9-fold, *p* < 0.001) and controls (miR-200c: 7.8-fold, *p* < 0.001; miR-141: 18.4-fold, *p* < 0.001) (Fig. 4b). There was no difference between localized breast cancers and controls, between tumor grades, or between ductal and lobular cancers (Fig. 4b and Additional file 1: Figure S7A-C).

To assess diagnostic potential, the sensitivity and specificity of plasma miR-200c and miR-141 were evaluated. ROC analysis predicted the capacity of plasma miR-200c and miR-141 to differentiate localized cases (Fig. 4c) from normal control cases or metastatic cases from localized cases (Fig. 4d). For localized cases versus controls, the area under curve (AUC) for plasma miR-200c and miR-141 was 0.557 (95% confidence intervals: 0.441, 0.672) and 0.582 (0.463, 0.702), respectively, but, for metastatic cases versus localized cases, the AUC for plasma miR-200c and miR-141 was higher at 0.770 (0.661, 0.880) and 0.678 (0.558, 0.799), respectively (Figs. 4c and d). These data reflect the patterns found in our mouse model (Fig. 3d). There were no significant differences between localized cases, metastatic cases, and controls in the levels of miR-200c and miR-141 in peripheral blood cells (Fig. 4e). Because hormone-negative breast cancers (ER/PR-negative) and HER2-positive breast cancers are more likely to reoccur and spread outside the breast tissue compared to ER/PR-positive breast cancers [47], the effect of ER/PR/HER2 status on plasma levels of miR-200c and miR-141 was evaluated. There were no significant differences among the subgroups according to receptor status (Fig. 4f).

### During breast cancer metastasis, plasma levels of miR-200c and miR-141 are likely derived from tumor cells

**Fig. 5:**
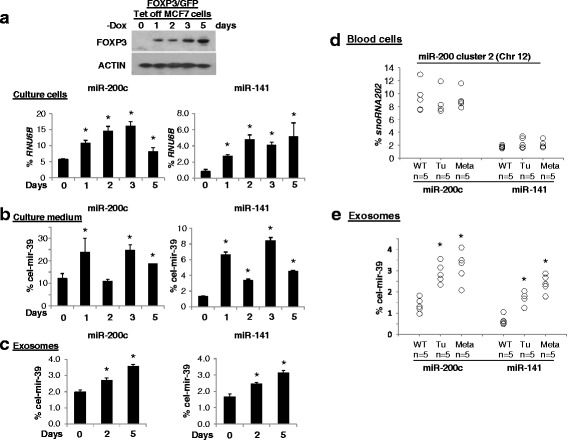
Levels of tumor-derived exosomal miR-200c and miR-141 in breast cancer cells and animals. FOXP3 protein expression was measured by western blotting in FOXP3/GFP-Tet-off MCF7 cells. *-Dox*, doxycycline removed from the culture medium. **a**-**c** Quantification of miR-200c and miR-141 (by quantitative (q)PCR) as a percentage of *RNU6B* or *cel-mir-39* expression in FOXP3/GFP-Tet-off MCF7 cells without Dox at 0, 1, 2, 3, and 5 days. Data are presented as means ± SD. *p < 0.05 vs. 0-h group (one-way analysis of variance (ANOVA) followed by the protected least significant difference test). **d** Levels of miR-200c and miR-141 in mouse blood cells (measured by qPCR) as a percentages of *snoRNA202* expression. **e** Quantification of levels of exosomal miR-200c and miR-141 in mouse plasma (by qPCR) as percentages of *cel-mir-39* expression. *WT* wild-type, *Tu* tumor, *Meta* metastasis. *p < 0.05 vs. no tumor group (one-way ANOVA followed by the protected least significant difference test). All experiments were repeated three times

Although, during breast cancer metastasis, there was differential expression of miR-200c and miR-141 in tumor cells (Figs. 3b and c) and plasma (Figs. 3d, 4a and b), whether miR-200c and miR-141 were released by tumor cells or blood cells [48] was not determined. To identify the source of plasma miR-200c and miR-141 and to characterize the transcriptional mechanisms regulating these molecules, an analysis of FOXP3/GFP Tet-off MCF7 cells was performed. After the removal of Dox, the levels of miR-200c and miR-141 in MCF7 cells, cell culture medium, and exosomes were measured before and after FOXP3 induction. At 0 to 5 days in culture without Dox, levels of miR-200c and miR-141 in tumor cells increased as FOXP3 was induced (Fig. 5a). Levels of extracellular miR-200c and miR-141 in the culture medium appeared to be increased, but there were intermittent increases and decreases (Fig. 5b). Levels of miR-200c and miR-141 in exosomes increased with time after FOXP3 induction (Fig. 5c). There were, however, no significant differences in the amounts of miR-200b, miR-200a, or miR-429 in MCF7 cells, cell culture medium, or exosomes (Additional file 1: Figure S8A–C). Since plasma miR-200c and miR-141 levels were elevated during tumor metastasis in *Foxp3* ^sf/+^ mice (Fig. 3d), the levels of miR-200 s in peripheral blood cells in *Foxp3* ^sf/+^ mice at 21 months of age were measured. However, in peripheral blood cells, no significant changes in the miR-200 s levels were evident (Figs. 5d and Additional file 1: Figure S9). These data suggest that the elevated plasma levels of miR-200c and miR-141 in these mice are not derived from peripheral blood cells.

Exosomes are nano-sized (50–150 nm) membrane vesicles secreted by most cell types. Notably, tumor cells secrete more than threefold more exosomes than normal cells [49]. To determine if miR-200c and miR-141 levels were changed in exosomes present in the plasma of mice, plasma was prepared from the blood of *Foxp3* ^sf/+^ mice at 2 years of age, and exosomes were extracted. Levels of exosomal miR-200c and miR-141 were higher (2.1/2.3-fold) in the mice with breast cancers, especially in those with tumor metastases (2.6/3.6-fold), than in mice without breast cancer (Fig. 5e). On the other hand, expression levels of miR-200c and miR-141 in exosomes, but not cell-free and exosome-free culture medium, were reduced in the *FOXP3*-knockdown MCF10A cells (Additional file 1: Figure S1C-D).

## Discussion

In the present study, we identified a FOXP3-KAT2B-miR-200c/141 transcriptional axis in breast cancer cells. However, the levels of miR-200c and miR-141 in tumor cells and in the circulation differed in mice, and differed in humans between patients with metastatic breast cancer, patients with localized breast cancer, and healthy female controls. The levels of miR-200c and miR-141 were low in primary tumor cells but were high in the circulation of patients with metastatic breast cancer. Further, our data suggested the cell origin of circulating miR-200c and miR-141 and their transcriptional regulation in cultured cells and during tumor progression in animal models of spontaneous breast cancer. Of note, miR-200c and miR-141 appeared to be released from breast cancer cells into the culture medium or into the circulation of mice during tumor progression.

Functional studies have found conflicting results on the role of miR-200 s in suppressing or promoting metastasis in different cellular contexts [11–14]. Likewise, early evidence showed increased expression levels of miR-200c and miR-141 in metastatic breast tumors [13], but recent studies revealed decreased expression of miR-200c in primary breast tumors with lymph node metastasis [50–52]. Our analysis of TCGA database identified downregulation of miR-200 s in primary breast cancer cells in patients with distant metastases. With the *Foxp3* ^*sf*/+^ spontaneous breast cancer mouse model, we observed downregulation of miR-200c and miR-141 in primary breast cancer cells, especially in mice with lung metastases. Thus, these data suggest that FOXP3-induced miR-200c/141 may function as metastatic suppressors at primary sites [11, 12, 18, 19] but as metastatic promotors at metastatic sites [13, 14].

The clinical significance of circulating miR-200 s in patients with breast cancer has been investigated in a few recent studies. In patients with breast cancer, increased levels of circulating miR-200 s may be a predictor of CTCs [21], metastasis up to 2 years prior to clinical diagnosis [22], and poor survival [23]. In the present study, we found increased levels of plasma miR-200c in patients with metastatic breast cancer who had no metastasis at diagnosis, as compared to those in patients with localized breast cancer. These findings are consistent with those observed during tumor metastasis in *Foxp3* ^sf/+^ mice. Since plasma miR-200c and miR-141 increase prior to metastasis, they may serve as biomarkers for early detection of tumor metastasis, which is supported by results of recent studies [5, 21, 22]. However, in both humans and mice, we found that miR-200c and miR-141 decrease in tumors but increase in the circulation, and are not changed in the blood cells. Of note, we also observed secretions of miR-200c and miR-141 in culture medium or in the circulation through exosomes in breast cancer cells and in animal models. Since expression levels of miR-200c and miR-141 in blood cells are not changed in humans or mice, tumor cells are likely to be the source, but future studies are needed to address this complex mechanism of action.

During tumor progression, some miRs are released from blood cells [48]. Here, we measured the levels of miR-200c and miR-141 in tumor cells, cell culture medium, and exosomes from FOXP3/GFP-Tet-off MCF7 cells. During FOXP3 induction, exosomal miR-200c and miR-141 were elevated, along with intracellular miR-200c and miR-141. Although extracellular miR-200c and miR-141 were also increased in cell culture medium, this increase was not dependent on intracellular miR-200c and miR-141. Thus, exosomes may be transporters for releasing or secreting miR-200c and miR-141 from tumor cells. In *Foxp3* ^sf/+^ mice, circulating miR-200c and miR-141 are unlikely to be released from blood cells. As circulating miR-200 s reflect the CTC status of patients with breast cancer [21], they may be released from CTCs through exosomes rather than from blood cells or primary tumor cells. In further studies, isolation of circulating CTCs and exosomes from mice or patients with breast cancer and comparison of expression levels of miR-200 s between CTCs and exosomes will address this hypothesis. A recent functional analysis revealed that extracellular vesicles containing miR-200c and miR-141 promote breast cancer cell metastasis through an enhanced MET transition [20]. This finding supports the idea that exosomal miR-200c and miR-141 are involved in metastasis of breast cancer and that they are biomarkers for early prediction of tumor metastasis.

Recent studies suggest that the miR-200c/141 cluster may act as a suppressor for the early stages of metastasis, but facilitate post-extravasation events, and that the miR-200b/a/429 cluster suppresses metastasis at all stages [13, 14, 53]. In addition, the promoter regions of the two clusters may be bound by different transcription factors. ZEB1 and ZEB2 both bind to the two miR-200 clusters, causing inhibition of the transcription of all miR-200 s; Sp1 binds the miR-200b/a/429 cluster [44, 54] and p53 binds the miR-200c/141 cluster [44, 55], leading to activation of transcription of miR200s. Likewise, direct binding of the KAT2B/P300/ZEB1 complex to the miR200c/141 promoter activates their transcription in cancer cells, but disruption of KAT2B and P300 interactions downregulates the promoter activity of miR-200c/141, indicating a direct functional regulation of miR200c/141 by the KAT2B/P300/ZEB1 complex in cancer cells [39]. In the present study, FOXP3, through direct binding of KAT2B to the miR-200c/141 cluster, leads to activation of transcription of miR200c/141. These data suggest the presence of a FOXP3-KAT2B-miR200c/141 axis in breast cancer cells and a differential mechanism of transcriptional regulation between the two miR-200 clusters.

## Conclusions

In breast cancer cells, miR-200c and miR-141 are regulated by a FOXP3-KAT2B axis, and circulating miR-200c and miR-141 are potential biomarkers for early detection of tumor metastasis. These observations will help in understanding how to predict metastasis of breast cancer and how to treat metastases.

## Additional files

Additional file 1: Table S1. Primer and siRNA sequence used in this study. **Figure S1.** Levels of miR-200c and miR-141 in normal immortalized human epithelial cell line MCF10A. **Figure S2.** Associations between expression of FOXP3 and miR-200 s in TCGA breast cancer samples. **Figure S3.** Potential binding signals of FOXP3 in the promoter regions of the miR-200 family and KAT2B gene. **Figure S4.** The transcriptional activity of miR-200b/a/429 on miR-200 cluster 1 after FOXP3 induction and KAT2B silencing in breast cancer cells. **Figure S5.** Association of expression levels of miR-200 s with ER/PR/HER2 status in breast cancer cells. **Figure S6.** Levels of plasma miR-200b, 200a, and 429 in Foxp3sf/+ female mice during tumor progression. **Figure S7.** Plasma levels of miR-141 and 200c in breast cancer patients with clinical parameters. **Figure S8.** Levels of miR-200b, 200a, and 429 in FOXP3-Tet-off MCF7 cells, culture medium, and exosomes. **Figure S9.** Expression of miR-200b, 200a, and 429 in blood cells of Foxp3sf/+ female mice. (DOCX 910 kb)

## Abbreviations

aCt: Adjusted cycle threshold
ANOVA: Analysis of variance
AUC: Area under the curve
ChIP: Chromatin immunoprecipitation
CTCs: Circulating tumor cells
DCIS: Ductal carcinoma in situ
Dox: Doxycycline
EMT: Epithelial-mesenchymal transition
ER: Estrogen receptor
HER2: Human epidermal growth factor receptor 2
MET: Mesenchymal-epithelial transition
miR: MicroRNA
PBS: Phosphate-buffered saline
PR: Progesterone receptor
qPCR: Quantitative PCR
ROC: Receiver operating characteristic
Sf: Scurfy
siRNA: Small interfering RNA
TCGA: The Cancer Genome Atlas
TIL: Tumor-infiltrating lymphocyte
UAB: University of Alabama at Birmingham
