# Supplementary material for: MicroRNA-200c and microRNA- 141 are regulated by a FOXP3-KAT2B axis and associated with tumor metastasis in breast cancer
Source: Breast Cancer Res. 2017 Jun 21;19:73. doi: 10.1186/s13058-017-0858-x (PMC5480201; doi:10.1186/s13058-017-0858-x)
Supplement: Supplementary file 1 — Primer and siRNA sequence used in this study. Figure S1. Levels of miR-200c and miR-141 in normal immortalized human epithelial cell line MCF10A. Figure S2. Associations between expression of FOXP3 and miR-200 s in TCGA breast cancer samples. Figure S3. Potential binding signals of FOXP3 in the promoter regions of the miR-200 family and KAT2B gene. Figure S4. The transcriptional activity of miR-200b/a/429 on miR-200 cluster 1 after FOXP3 induction and KAT2B silencing in breast cancer cells. Figure S5. Association of expression levels of miR-200 s with ER/PR/HER2 status in breast cancer cells. Figure S6. Levels of plasma miR-200b, 200a, and 429 in Foxp3sf/+ female mice during tumor progression. Figure S7. Plasma levels of miR-141 and 200c in breast cancer patients with clinical parameters. Figure S8. Levels of miR-200b, 200a, and 429 in FOXP3-Tet-off MCF7 cells, culture medium, and exosomes. Figure S9. Expression of miR-200b, 200a, and 429 in blood cells of Foxp3sf/+ female mice. (DOCX 910 kb) [file 13058_2017_858_MOESM1_ESM.docx]

**Supplemental Data**

**MicroRNA-200c and 141 are regulated by a FOXP3-KAT2B axis and associated with tumor metastasis in breast cancer**

Guangxin Zhang, Wei Zhang, Bingjin Li, Erica Stringer-Reasor, Chengjing Chu, Liyan Sun, Sejong Bae, Dongquan Chen, Shi Wei, Kenneth Jiao, Wei-Hsiung Yang, Ranji Cui, Runhua Liu and Lizhong Wang

| **Table S1. Primer and siRNA sequence used in this study** | |
| --- | --- |
| **Primer Name** | **Sequence** |
| Human KAT2B-realtime-F | CCGACCTGCAGCAAATAATT |
| Human KAT2B-realtime-R | TTCCCAGGAGTCTGTTCATTT |
| Human PITX2-realtime-F | CACTTTCCAGAGGAACCGCTA |
| Human PITX2-realtime-R | AGTGTTCACATCGGACACCA |
| Human KLF5-realtime-F | CACAAAACATCCAACCTGTCA |
| Human KLF5-realtime-R | TGAGTCCTCAGGTGAGCTTTT |
| Human MUC1-realtime-F | TTCACCACCACCATGACACC |
| Human MUC1-realtime-R | TTCAGAGACAGCCAGGAGAAA |
| Human TP53-realtime-F | TGCCTGATACAGATGCTACTTGA |
| Human TP53-realtime-R | TTGAGTTCCAAGGCCTCATT |
| Human GAPDH-realtime-F | CCCCTTCATTGACCTCAACTACAT |
| Human GAPDH-realtime-R | CGCTCCTGGAAGATGGTGA |
| has and mmu-miR-200c | CCCTAATACTGCCGGGTAATGATGGA |
| has and mmu-miR-141 | CCCTAACACTGTCTGGTAAAGATGG |
| has and mmu-miR-200b | CCCTAATACTGCCTGGTAATGATGA |
| has and mmu-miR-200a | CCCTAACACTGTCTGGTAACGATGT |
| has-miR-429 | CCCTAATACTGTCTGGTAAAACCGT |
| mmu-miR-429 | CCCTAATACTGTCTGGTAATGCCGT |
| has-miR-200c/141-ChIPseq-F (-20kb) | CAATCCACTAATTCCTTGCG |
| has-miR-200c/141-ChIPseq-R (-20kb) | TTGCAGCATCTTACCCATGT |
| has-miR-200c/141-ChIPseq-F (-4.5kb) | CAGAGAATAGGGGAATGGGAA |
| has-miR-200c/141-ChIPseq-R (-4.5kb) | TAGATTCAGCCGACTTGCC |
| has-miR-200c/141-ChIPseq-F (-4.2kb) | TCTCCCTGAGGTCTGTTTGC |
| has-miR-200c/141-ChIPseq-R (-4.2kb) | AAGGATAGCCCTGAGACAGCA |
| has-miR-200c/141-ChIPseq-F (-2.4kb) | AATTTAAATGGCTGCATCCCC |
| has-miR-200c/141-ChIPseq-R (-2.4kb) | GCAAGCAAAGGGCTCAGA |
| has-miR-200c/141-ChIPseq-F (-1.3kb) | TGGAACTTAGGAGGCTGGTC |
| has-miR-200c/141-ChIPseq-R (-1.3kb) | TATTGGGTTGCTCAATCCCT |
| has-miR-200c/141-ChIPseq-F (+0.23kb) | AGCTGAGAGCGTTGCACAA |
| has-miR-200c/141-ChIPseq-R (+0.23kb) | ATCCACCAGGTGACAAATCG |
| has-miR-200c/141-ChIPseq-F (+0.7kb) | AATCACTAGGCAAAGGGGAA |
| has-miR-200c/141-ChIPseq-R (+0.7kb) | TTATTTCATGCTCCCAAGGC |
| has-miR-200c/141-ChIPseq-F (+1.2kb) | AAGGCCTGTCTTGGGAAGATA |
| has-miR-200c/141-ChIPseq-R (+1.2kb) | ATTTCTCAGTCACCAGGCGT |
| Human KAT2B siRNA-1 | GCATGTCCATTAGCTATTTCA |
| Human KAT2B siRNA-2 | GTCTTGGGATTCCAGTTTAGT |
| Human PITX2 siRNA-1 | GAAGGTCGTGGGCACTAAAGA |
| Human PITX2 siRNA-2 | GAAACCACTGAATCAAAGAGA |


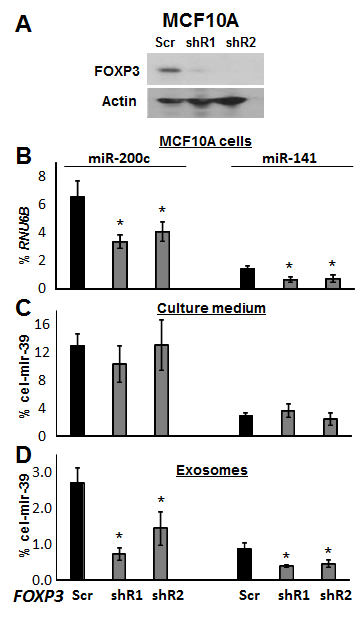


**Figure S1. Levels of** **miR-200c and 141 in normal immortalized human epithelial cell line MCF10A. A**, Western blot analyses showing protein expressions of FOXP3 in Scr or shRNA-transfected cells. **B**, quantification (by TaqMan miR assay) of miR-200c and 141 as percentages of *RNU6B* expression in MCF10A cells with and without FOXP3. **C**, quantification of levels of miR-200c and 141 (by nest-quantitative PCR) as percentages of cel-mir-39 expression in cell- and exosome-free culture medium with and without FOXP3. **D**, quantification (by nest-quantitative PCR) of levels of exosomal miR-200c and 141 as percentages of cel-mir-39 expression in exosomes with and without FOXP3. Data are presented as means ± SD. * *p* < 0.05 vs. Scr group (two-tailed t test). Scr, scramble; shRNA, short hairpin RNA. All experiments were repeated three times.


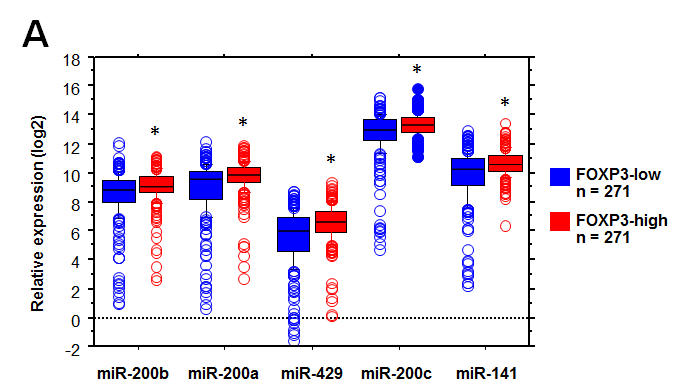


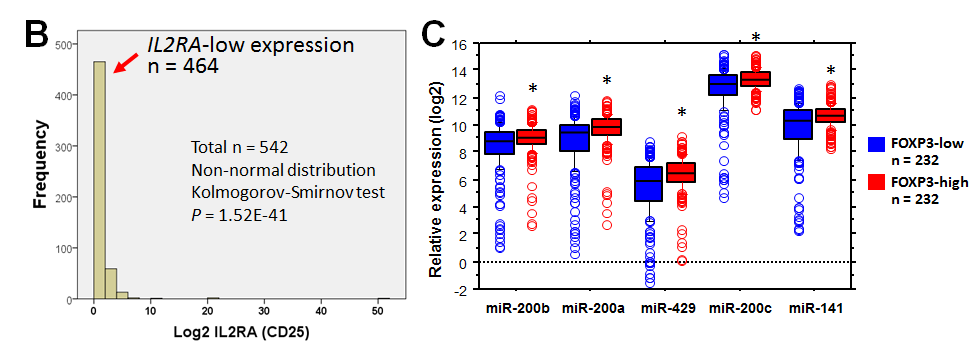


**Figure S2.** **Associations between expression of *FOXP3* and miR-200s in TCGA breast cancer samples. A,** expression of miR-200 family members (miR-200 cluster 1: miR-200b/a/429; miR-200 cluster 2: miR-200c/141) in human breast cancer samples from 542 patients listed in the NCI TCGA. Values for *FOXP3*^low^ and *FOXP3*^high^ were divided by a median of *FOXP3* expression. All data were expressed on a log2 basis with a normal distribution for each group. * *p* < 0.001 in *FOXP3*^high^ *vs*. *FOXP3*^low^, two-tailed *t* test. **B**, non-normal distribution of *IL2RA* (*CD25*) expression in 542 TCGA breast cancer samples. Since FOXP3^+^ tumor-infiltrating regulatory T cells may contribute to the expression of *FOXP3* in tumors, we conducted a stratification of patients by *IL2RA* expression, which is specifically expressed in regulatory T cells. A non-normal distribution of *IL2RA* expression was evident for all tumors, including 464 with low-expression (0.08-1.98 with normal distribution) and 78 with over-expression (2.02-51.61 with non-normal distribution). **C**, expression of miR-200 family members in *IL2RA*-low breast cancers from 464 patients in the NCI TCGA. To avoid the effect of FOXP3^+^ regulatory T cells, we selected the 464 *IL2RA*-low tumors to address the association between expression of *FOXP3* and miR-200s in human breast cancers. The *FOXP3*^low^ and *FOXP3*^high^ subgroups were divided by a median of *FOXP3* expression. All data were expressed on a log2 basis, with a normal distribution for each group. * *p* < 0.001 in *FOXP3*^high^ *vs*. *FOXP3*^low^, two-tailed *t* test.


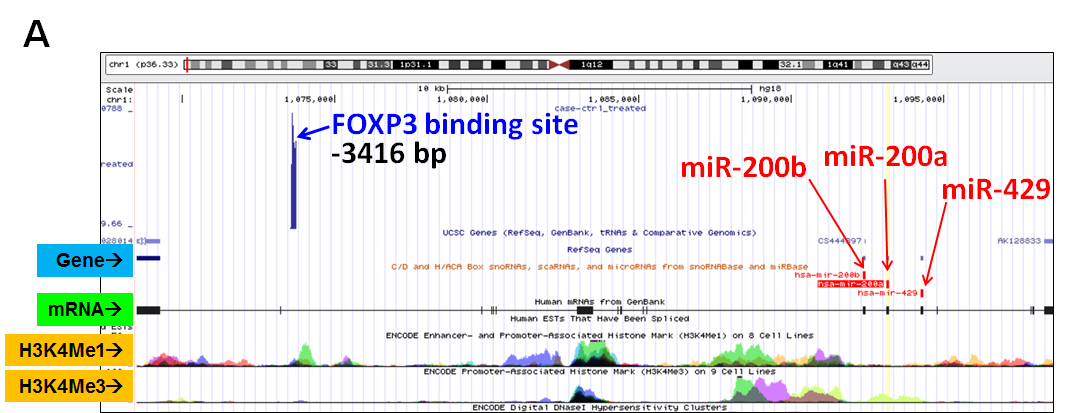


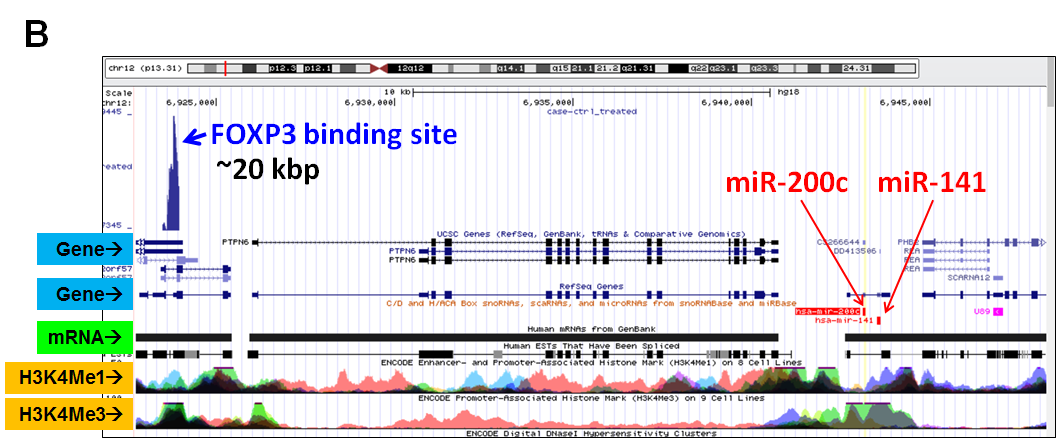


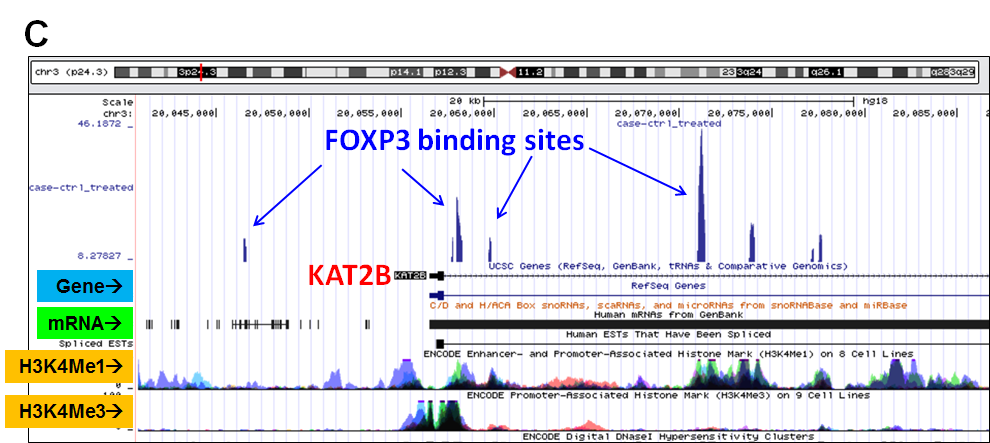


**Figure S3. Potential binding signals of FOXP3 in the promoter regions of the miR-200 family and *KAT2B* gene.** ChIP-seq data as custom tracks within the UCSC genome browser were used to identify the FOXP3 binding sites by ChIP-seq peak detection in promoter regions of miR-200 cluster 1 from human chromosome 1 (**A**), cluster 2 from human chromosome 12 (**B**), and the *KAT2B* gene from human chromosome 3 (**C**). At the top of each panel, the red vertical line within the chromosome indicates the localization of miR-200s or the *KAT2B* gene. The blue arrows indicate the FOXP3 binding site, and the red arrows indicate the miR-200 family members. Chr, chromosome; bp, base pair.


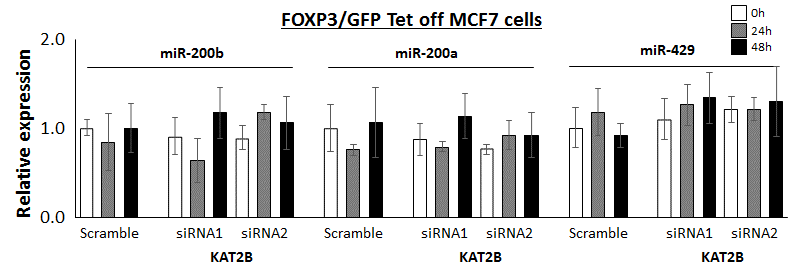


**Figure S4. The transcriptional activity of miR-200b/a/429 on miR-200 cluster 1 after *FOXP3* induction and *KAT2B* silencing in breast cancer cells.** The relative quantification of miR-200b/a/429 (by qPCR) as percentages of *RNU6B* in FOXP3/GFP-Tet-off MCF7 cells with scramble or siRNAs of *KAT2B* at 0, 24, and 48 hours. All data for each group were normalized to the scramble control at 0 hour. Data are presented as the means ± SD of triplicates. All *p* > 0.05 vs. 0-hour group (one-way ANOVA followed by protected least-significant difference test). All experiments were repeated three times.


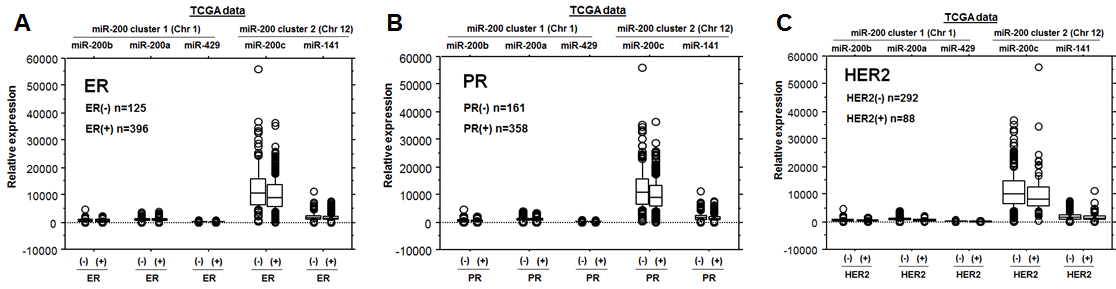


**Figure S5. Association of expression levels of miR-200s with ER/PR/HER2 status in breast cancer cells**. The expressions of miR-200s in breast cancer cells were analyzed for patients with breast cancers from the NCI TCGA. The subgroups were divided by (**A**) ER status, (**B**) ER status, and (**C**) HER2 status. The box-and-whisker plots of expression levels of miR-200s are presented as relative values for breast cancer patients with ER, PR, and HER2 status. In samples with normal distributions, the means of the variables were compared by use of a two-tailed *t* test between two groups. In samples with non-normal distributions, the medians of the variables between two groups were compared with a Mann-Whitney test. Between subgroups, no significant difference in expression levels of miR-200s was found for breast cancer cells. ER, estrogen receptor; PR, progesterone receptor; HER2, human epidermal growth factor receptor 2.


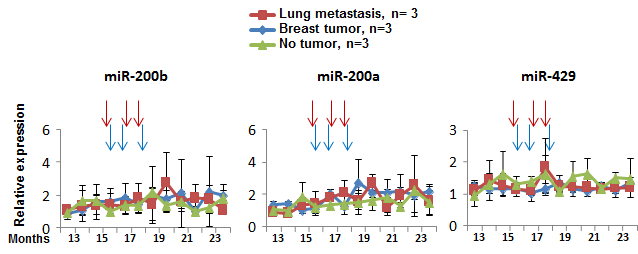


**Figure S6. Levels of plasma miR-200b, 200a, and 429 in *Foxp3*^sf/+^ female mice during tumor progression.** The relative expression was determined by nest-qPCR using the 2^-∆Ct^ against the Ct value at 1 year of age (* *p* < 0.05 vs. no tumor group; two-way ANOVA). The time points of tumor development (determined by visual observation) are indicated by vertical arrows. All experiments were repeated three times.


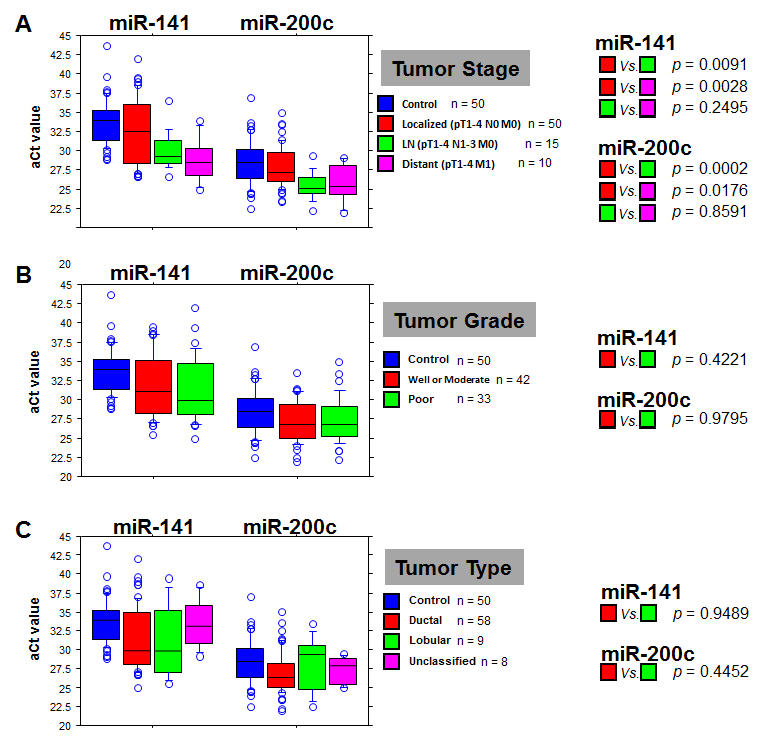


**Figure S7. Plasma levels of miR-141 and 200c in breast cancer patients with clinical parameters.** Box-and-whisker plots of plasma miR-200c and 141 levels are presented as adjusted PCR cycle threshold (aCt) values for patients with tumor stages (**A**), tumor grades (**B**), and tumor types (**C**). The aCt value of each miR was adjusted by the spiked-in control, cel-miR-39. In samples with normal distributions, the means of the variables were compared by use of a two-tailed t test between two groups. In samples with non-normal distributions, the medians of the variable between two groups were compared with a Mann-Whitney test. Local, localized breast cancer; LN, lymph node metastasis; Meta, distant metastasis, Well, well-differentiated tumor; Moderate, moderately differentiated tumor; Poor, poorly differentiated tumor; Ductal, invasive lobular carcinoma; Lobular, invasive ductal carcinoma; Unclassified, unclassified tumor type. All experiments were repeated three times.

**Figure S8. Levels of miR-200b, 200a, and 429 in FOXP3-Tet-off MCF7 cells, culture medium, and exosomes.** Measurements of miR-200b, 200a, and 429 were determined by qPCR; values were percentages of *RNU6B* or *cel-mir-39* expression. Cells (**A**), culture medium (**B**), and exosomes (**C**) in the FOXP3-Tet-off MCF7 cells without Dox at 0, 1, 2, 3, and 5 days. Data are presented as means ± SD. * *p* < 0.05 (one-way ANOVA followed by protected least significant difference test for differences between groups). All experiments were repeated three times.


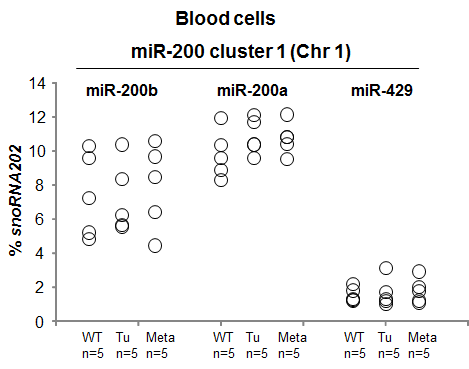


**Figure S9. Expression of miR-200b, 200a, and 429 in blood cells of *Foxp3*^sf/+^ female mice.** Measurements of miR-200b, 200a, and 429 were by qPCR; values were expressed as percentages of *snoRNA202* expression. WT, wild-type; Tu, tumor; Meta, metastasis; Chr, chromosome. No significant difference was found among the WT, Tu, and Meta groups (one-way ANOVA followed by protected least significant difference test for differences between groups). All experiments were repeated three times.
